# Exercise Testing, Physical Training and Fatigue in Patients with Mitochondrial Myopathy Related to mtDNA Mutations

**DOI:** 10.3390/jcm10081796

**Published:** 2021-04-20

**Authors:** Tina D. Jeppesen, Karen L. Madsen, Nanna S. Poulsen, Nicoline Løkken, John Vissing

**Affiliations:** Copenhagen Neuromuscular Center, Department of Neurology, Rigshospitalet University Hospital of Copenhagen, 2100 Copenhagen, Denmark; karen.lindhardt.madsen@regionh.dk (K.L.M.); nanna.scharff.nielsen.01@regionh.dk (N.S.P.); nicoline.loekken@regionh.dk (N.L.); john.vissing@regionh.dk (J.V.)

**Keywords:** mitochondrial myopathy, mtDNA mutation, exercise testing, fatigue

## Abstract

Mutations in mitochondrial DNA (mtDNA) cause disruption of the oxidative phosphorylation chain and impair energy production in cells throughout the human body. Primary mitochondrial disorders due to mtDNA mutations can present with symptoms from adult-onset mono-organ affection to death in infancy due to multi-organ involvement. The heterogeneous phenotypes that patients with a mutation of mtDNA can present with are thought, at least to some extent, to be a result of differences in mtDNA mutation load among patients and even among tissues in the individual. The most common symptom in patients with mitochondrial myopathy (MM) is exercise intolerance. Since mitochondrial function can be assessed directly in skeletal muscle, exercise studies can be used to elucidate the physiological consequences of defective mitochondria due to mtDNA mutations. Moreover, exercise tests have been developed for diagnostic purposes for mitochondrial myopathy. In this review, we present the rationale for exercise testing of patients with MM due to mutations in mtDNA, evaluate the diagnostic yield of exercise tests for MM and touch upon how exercise tests can be used as tools for follow-up to assess disease course or effects of treatment interventions.

## 1. Introduction

Primary mitochondrial disease, due to mutations in mitochondrial DNA (mtDNA), is characterized by high variability of clinical presentation. Symptoms range from adult-onset monosymptomatic myopathy to multi-organ affection in infancy. In patients with mtDNA mutations, both wild-type and mutated mtDNA can exist in almost all cells, and the oxidative capacity of each tissue correlates directly with the load of mutated mtDNA copies [1]. Thus, the heterogeneous clinical presentation may be related, at least in part, besides affected gene and mutation type, to differences in mtDNA mutation load among tissues [2,3].

**Figure 1:**
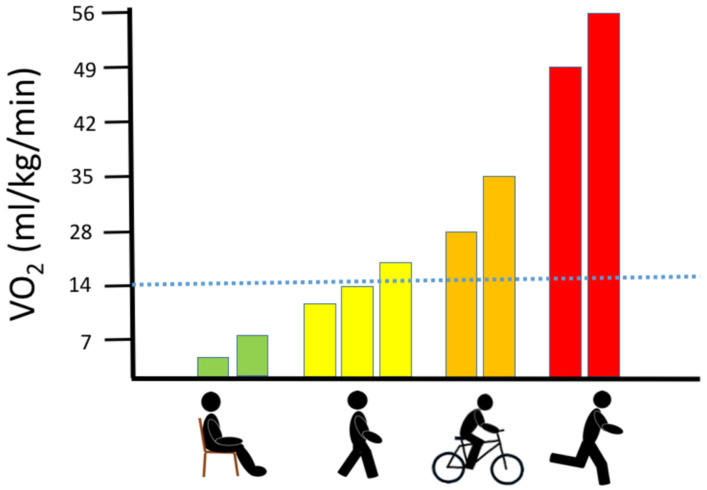
Association between oxygen uptake and different physical activities.

In patients with mitochondrial myopathy (MM), the most prominent clinical hallmarks are exercise intolerance [4,5] and premature fatigue [6]. The physical capacity of patients with MM is often so low that even trivial physical activities, such as walking, and household activities, such as cleaning and cooking, are troublesome (Figure 1). The pronounced involvement of skeletal muscle is thought to be related to the high mtDNA mutation load in skeletal muscle [3,7,8] combined with a high oxygen demand that increases up to 100-fold from rest to exercise, which is unmatched by any other tissue [6,9].

The graph demonstrates the range of oxygen demand (visualized as the corresponding oxygen uptake (VO_2_)) during no activity (sitting), walking (slow to brisk walking speed), jogging and running at maximal speed. The dotted blue line denotes the maximal oxidative capacity found in patients with high levels of mtDNA mutation load in skeletal muscle [7].

Oxidative capacity in skeletal muscle can be evaluated with exercise tests. Since oxidative capacity indirectly is a measure of mitochondrial function, exercise studies have been used to elucidate the physiological consequences of defective mitochondria in patients with mtDNA mutations [2,3,7,8,10,11,12,13,14,15,16,17,18,19,20,21,22,23,24,25,26,27,28]. Diagnosing patients with obvious phenotypes related to mitochondrial disease may seem straightforward and directly lead to gene sequencing of putative mtDNA aberrations. However, in patients complaining of pure exercise intolerance, monosymptomatic fatigue or abnormal dyspnea during light exercise, mitochondrial disease may not be obvious [5,29,30,31,32,33], and instead be mistaken for a cardiac or pulmonary disease, or even non-organic disorder. In some of these cases, exercise testing has been suggested as helpful in the diagnostic work-up of patients suspected of mitochondrial disorder. In this review, we present the rationale for exercise testing of patients with MM related to mutations in mtDNA, discuss the diagnostic yield of these tests and how these tests may be used as tool to assess disease course or effects of treatment interventions.

## 2. Oxygen Delivery during Exercise in Healthy Skeletal Muscle

### 2.1. Oxygen Delivery: From Air to Contracting Muscle

The first path of oxygen delivery from air to the muscle is oxygen passing through the lungs to the blood. Pulmonary oxygen uptake is tightly coupled to demand and is regulated via the pulmonary ventilation rate that increases linearly with exercise intensity until maximal oxygen uptake is reached [34,35,36]. At the point of maximal oxygen uptake (VO_2max_), the ventilation rate actually exceeds that of oxygen uptake, and for a short while, subjects are hyperventilating until oxygen uptake reaches a plateau. When oxygen has passed to the capillaries in the lungs, it is linked to blood hemoglobin and the circulation is driven by the heart. Cardiac output (heart rate × stroke volume) determines the amount of oxygen that is delivered to contracting skeletal muscle. There is a 5:1 ratio between delivery of blood and muscle utilization of oxygen irrespective of age, gender and exercise capacity [6,37,38]. When oxygen is delivered to a contracting muscle, it is used for the oxidation of substrates in the mitochondria.

### 2.2. Oxygen Delivery: Neural Regulation

**Figure 2:**
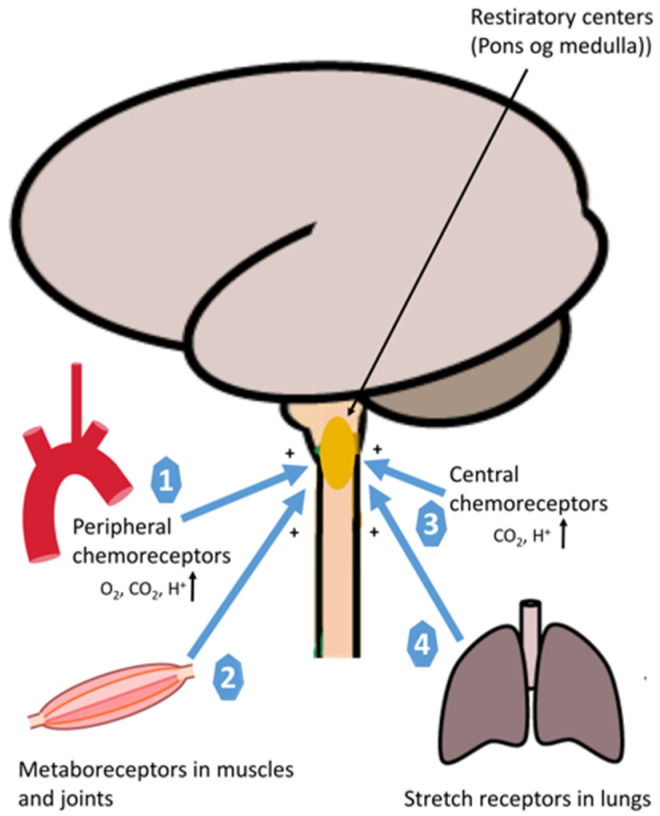
An illustration of neural regulation of ventilation during exercise. Peripheral chemoreceptors monitor the partial pressure of arterial O_2_ in blood in response to acidosis (H^+^) and carbon dioxide (CO_2_) (1), Metaboreceptors stimulate breathing during exercise (2), Central chemoreceptors monitor the partial pressure of arterial O_2_ in blood, pH, and carbon dioxide (CO_2_) (3), Mechanoreceptors via spindles in the respiratory muscles (intercostal and diaphragm) measure the muscle length and increase the motor discharge (4).

**Figure 3:**
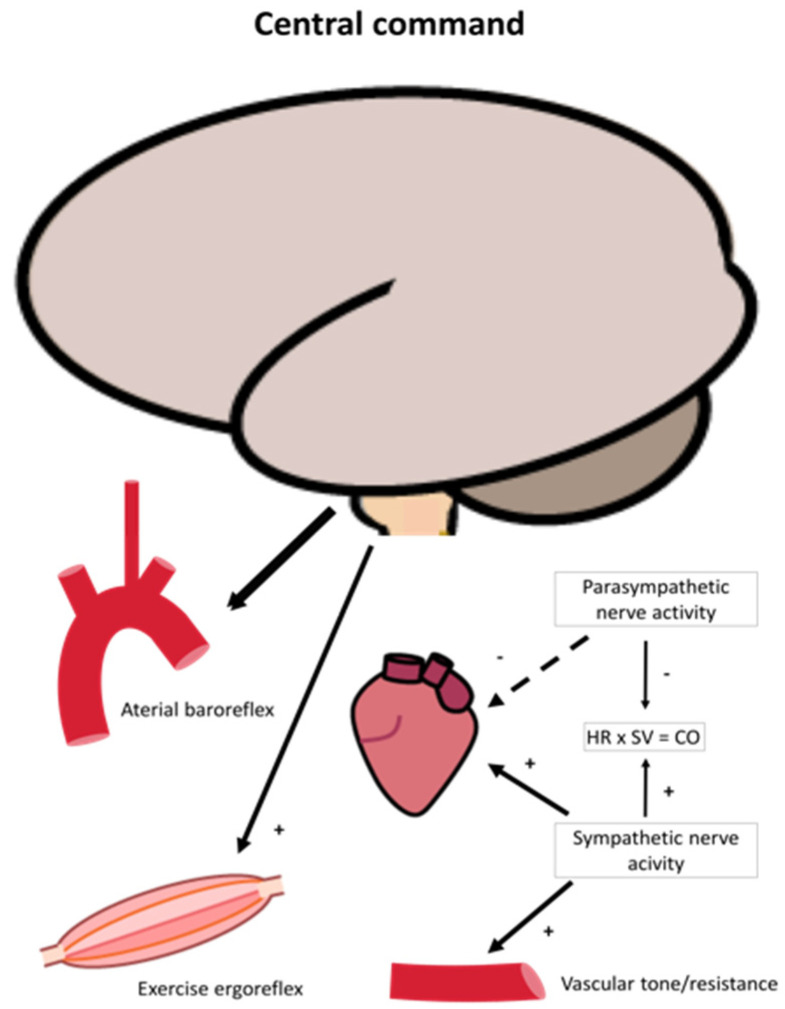
An illustration of neural regulation of cardiovascular control during exercise. Neural command originates from the brain and reset blood pressure and heart rate through arterial baroreflex and act through ergoreflex that in conjunction modulate sympathetic and parasympathetic nerve activity during exercise. Parasympathetic drive is diminished resulting in higher sympathetic activity, increasing heart rate and arterial contractility and resistance/capacitance of the vessels. As a result, heart rate (HR) and stroke volume (SV), and thus cardiac output increases.

**Figure 4:**
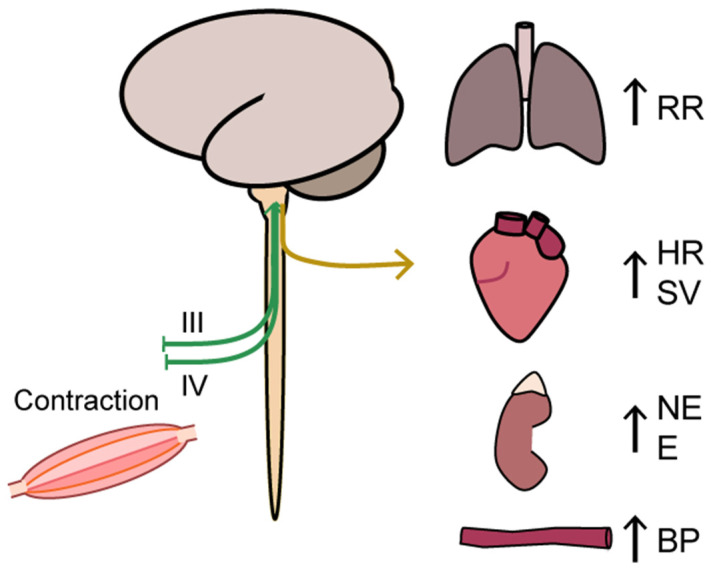
An illustration of the ergoreflex via a central command to the muscle that signals the muscle to contract the contraction activates the ergoreflex through mechanoreceptors (group III afferents) and metaboreceptors (group IV afferents), which in a feedback manner stimulate autonomic functions and ventilation rate. BP: blood pressure, E: epinephrine, HR: heart rate, NE: norepinephrine, VR: ventilation rate, SV: stroke volume.

An extensive description of the neural regulation of ventilation rate and cardiovascular response to exercise is not the scope of this review but is briefly described in the following section. The rate of pulmonary ventilation increases rapidly from the first breath at onset of exercise linearly with exercise intensity until the VE threshold is reached [39,40,41]. Ventilation rate is tightly regulated by direct feedback to the respiratory muscles from the central nervous system (respiration center in pons and medulla oblongata), and the signal is again tightly regulated by a complex set of sensory input systems, i.e., metaboreceptors in skeletal muscle, peripheral chemoreceptors that respond to changes in oxygen, carbon dioxide, acidosis (H^+^) and central chemoreceptors that senses changes in pH, oxygen and carbon dioxide in the brain [35,42]. During light to moderate exercise, the main driving parameter of ventilation rate is carbon dioxide, while the parameters driving ventilation above the anaerobic threshold are mainly pH and H^+^ [43] (Figure 2). The cardiovascular response to exercise is managed through a similar tight interaction between central and peripheral neural efferent and afferent systems that ensure that cardiac output matches oxygen demand in skeletal muscle. These systems include central command, arterial baroreflex and the exercise pressor reflex/ergoreflex (Figure 3) [42,44,45,46]. Blood pressure is regulated by continues changes in heart rate, stroke volume and peripheral resistance via the arterial baroreflex, i.e., afferent fibers that originates in the carotid sinus and aortic arch [47,48]. At onset of exercise, this level is reset if needed during exercise [49]. The central command consists of signals from the motor cortex or subcortical nuclei that recruit skeletal motor units and modulate the sympathetic and parasympathetic control through the brainstem [50] together adjust blood flow to exercising muscle through changes in cardiac output, heart rate and blood pressure [50] (Figure 3). With the onset of exercise, the central nervous system generates a sympathoadrenal response that reduces parasympathetic activity to the heart and resets the arterial baroreflex, leading to increased blood and vascular pressure (Figure 3). The muscle contraction further activates the ergoreflex (Figure 4), which signals through the afferent nerves of group III and IV that further drives ventilation rate, heart rate and blood pressure through activation of the sympathetic system [51]. The vasoconstriction that ensures sufficient rise in blood pressure, which is driven by norepinephrine release, is blocked by functional sympatholysis in contracting muscle [6,37]. This allows redirection of blood from other organs, including redirection of blood from resting to contracting skeletal muscle fibers. Studies have shown that the red blood cells play an important role acting as local vasodilators and may even be key mediators of functional sympatholysis through off-loading of ATP in response to oxygen release when passing through the capillaries of contracting muscle [11,52,53,54,55,56]. Moreover, ATP activates nerve endings of group III and IV thin fiber afferents through P2 receptors during exercise and contribute to exercise ergoreflexes by sensitizing group III (mechanoreceptors) and stimulating group IV (metaboreceptors) [57,58,59] (Figure 4).

### 2.3. Oxygen Delivery: Rate Limiting Step

VO_2max_ is synonymous with maximal exercise capacity in healthy subjects [34,60,61,62,63]. This is so oxidation of adenosine di-phosphate to adenosine-tri-phosphate in skeletal muscle is the final biochemical bottleneck for contraction of muscle fibers. Many studies have investigated if the final step, the mitochondria, might be of some influence on peak exercise performance, but in healthy subjects, cardiac output is considered to be the main bottleneck and rate-limiting step for oxygen delivery and thus ATP production to contracting skeletal muscle fibers [9,38,62].

## 3. Oxygen Consumption during Exercise in Healthy Muscle

Mitochondria are responsible for oxidation of substrates. This allows a higher yield of ATP per molecule of glucose (three versus 37 ATP per glucose molecules) and allows humans to utilize free fatty acid (FFA) as fuel for muscle work [64,65]. In fact, until exercise intensity reaches 50% of VO_2max_, the primary fuel for muscle is FFA. During the first 30 to 60 s of exercise, anaerobic glycogenolysis and glycolysis is activated maximally allowing the initial ATP production that is needed for muscle contraction until FFA are being oxidized and glucose is being released from liver. This results in a spike in lactate level during the first five minutes of exercise. After a few minutes, the limb blood flow level and thus delivery of glucose and FFA from the liver is sufficient to meet the energy demand of the contracting myofibers. From this point, exercise intensity and duration determines the fraction of choice of substrate, i.e., FFA being the primary fuel used to sustain exercise, which reaches a maximum at exercise intensity of 65% of VO_2max_ [64]. Until this point, there is a balance between lactate production and clearance [66]. At 65% of VO_2max_, also denoted as the anaerobic threshold, there is a net lactate increase that increases linearly with increasing exercise intensity until the maximal carbohydrate oxidation rate is reached at 85% VO_2max_, and from this point the main source of ATP is through anaerobic metabolism [43,67].

## 4. Exercise Testing

### 4.1. Whole-Body Exercise

#### 4.1.1. Whole-Body Exercise: Maximal Oxygen Uptake (VO_2max_)

Maximal oxygen uptake (VO_2max_) is studied by performing whole-body exercise using a protocol of incremental exercise to exhaustion and by measurements of oxygen uptake and carbon dioxide production using the Douglas bag or the breath-by-breath metabolic cart method [68]. Cycling exercise on a stationary bike is more feasible in patients with MM compared to treadmill exercise, which can be difficult to perform for patients with ataxia, myoclonia and other gait disabilities often associated with mitochondrial disease, and perhaps therefore, cycle testing is the most commonly used exercise modality. It is well-established that patients with MM often have a low VO_2max_ [2,7], but the spectrum of VO_2max_ varies substantially, i.e., from near-normal to levels below what is required to sustain daily activities such as climbing stairs and cleaning. Studies have demonstrated that VO_2max_ correlates with the mtDNA mutation load in exercising muscle irrespective of mutation type [2,3,7,10], indicating that the mutation load, rather than the genotype, determines the oxidative capacity of skeletal muscle of MM. Thus, measurement of VO_2max_ via cycle ergometry is a non-invasive and effective method to assess oxidative capacity in skeletal muscle of MM.

#### 4.1.2. Whole-Body Exercise: Ventilation Rate

Hyperventilation and exertional dyspnea in patients with metabolic myopathies were first described in the early 1960s [69] and several studies have since demonstrated an abnormally high ventilation rate in patients with MM during exercise [13,18,19,31,33,70,71], and, in some MM patients, dyspnea may even be the most prominent complaint [31,33,72]. Interestingly, hyperventilation during exercise occurs despite a high oxygen content in arterial blood in patients with MM, indicating that the regulation of ventilation rate in relation to oxygen uptake is skewed [7,11,13]. Thus, in patients with MM, the ventilation to VO_2max_ ratio is higher than in healthy individuals, and the ratio in fact correlates directly with mtDNA mutation load in skeletal muscle [7,18]. It is yet unknown what drives the exaggerated ventilation rate during exercise in patients with MM. Changes in arterial CO_2_, H^+^, potassium, catecholamines, increased chemosensitivity, skeletal muscle afferent feedback, central command and cardiac afferent feedback have all been proposed to be potentially important mechanisms controlling ventilation rate during exercise [34,35,36,42,44,73]. Thus, dysfunction of these parameters could all mediate the exertional dyspnea found in MM patients. However, studies have argued that since ventilation rate has been shown to be tightly coupled to metabolic rate, the humoral mechanism seems to be the only mediator able to drive the neural feedback or feedforward mechanisms that is able to respond fast enough to adjust ventilation rate during exercise [44]. Thus, one important parameter that has been suggested as a mediator of hyperventilation is the lactic acidosis driven by impaired oxidative capacity in MM patients. The argument is that ventilation rate increases proportionally more than VCO_2_ when reaching maximal exercise capacity, which is thought to be driven by metabolic acidosis [34,35,36,44]. However, the only study that has investigated this, argued that given that the ventilation rate returns to normal at end-exercise despite a high post-exercise lactate level, indicates that the exertional dyspnea is not driven directly by the lactate level in MM patients [18]. Instead, hyperventilation has been coupled with increased ergoreflex sensitivity, which, at least in part, could be an important mediator of the exaggerated ventilation relative to workload that is seen in patients with MM [74]. The ergoreflex is a neuromuscular reflex that drives ventilation and sympathetic outflow during increasing exercise intensity [75] and is described more in detail in Section 4.1.4.

#### 4.1.3. Whole-Body Exercise: Oxygen Delivery and Extraction

Along with an increased ventilation rate, patients with MM have a hyperkinetic circulatory response to exercise [7,11,13,23,69,76] McCoy. By measuring the blood flow to exercising muscle with Doppler technique, we have shown that the higher cardiac output translates into blood flow levels that can be up to two-fold higher in patients with severely impaired oxidative capacity (range 0.1–0.3 L/min/Watt) compared to age- and gender-matched healthy subjects [11]. This hyperkinetic response translates into an almost arterialized level of oxygen in venous blood from contracting muscle. Interestingly, like VO_2max_ and ventilation rate, cardiac output and the level of oxygen extraction has been shown to correlate directly with the level of mtDNA mutation in contracting skeletal muscle [7,10], again indicating that the level of mtDNA mutation determines oxygen extraction and the level of blood flow to contracting muscle rather than the genotype. Thus, measurement of cardiac output, arterial oxygen content and extraction during exercise can be an important tool in determination of the level of oxidative capacity in patients with MM. Moreover, the findings of hyperkinetic response in MM indicates that the mitochondrial extraction of oxygen rather than the delivery is the limiting factor for maximal exercise, at least in patients with severely impaired oxidative capacity due to mtDNA mutations, which is in contrast to healthy persons, in whom mitochondrial oxidative capacity has been demonstrated to be in excess of oxygen delivery [77]. Interestingly, a study demonstrated that the absolute peak-exercise cardiac output seemed to be reduced in patients with mtDNA mutation, even patients with mild to moderate muscle affection. Authors interestingly demonstrated that this reduction was induced by reduced stroke-volume [78].

#### 4.1.4. Whole-Body Exercise: Autonomic Nervous System Regulation

**Figure 5:**
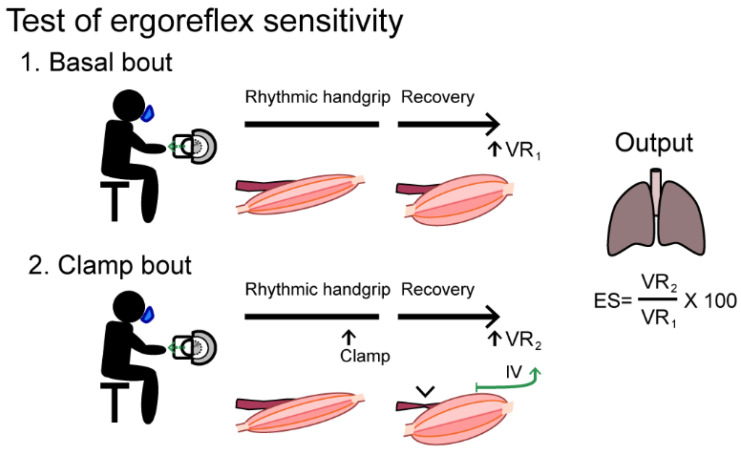
A handgrip test protocol to asses ergoreflex sensitivity.

During exercise, the level of circulating epinephrine and norepinephrine relative to workload is increased in MM patients [13,76,79,80,81,82]. This has introduced the idea that patients with MM may have an autonomic dysfunction causing the exaggerated circulatory and ventilatory response to exercise. Whether the exaggerated sympathetic response is related to dysfunction in central command (feedforward mechanisms) or peripheral nervous system regulation (feedback mechanisms) is yet unknown. In a study where central versus peripheral regulation of ventilatory response to exercise was examined in patients with MM, the authors argued that the feedforward mechanisms and activation of cortical and spinal motor neurons are not exaggerated [18]. Instead, the exaggerated norepinephrine and epinephrine levels could be related to ergoreflex hypersensitivity [74]. The ergoreflex sensitivity can be measured as the ratio of the post-exercise ventilation rate after circulatory clamp of one extremity vs post-exercise ventilation rate without clamp (Figure 5). Piepoli et al., demonstrated a two-fold higher ergoreflex sensitivity in patients with MM compared to healthy individuals. Interestingly, the study indicated that the level of ergoreflex sensitivity could predict the degree of cardiac affection related to mitochondrial disease (fibrosis, increased cardiac enzymes in plasma, decreased cardiac ejection fraction) and could potentially serve as risk stratification tool in relation to the often-overlooked cardiac involvement in patients with MM.

A mediator of the increased ergoreflex sensitivity or up-regulation could be increased circulating ATP released from circulating hemoglobin. We found evidence that red blood cells release increased amounts of the vasodilator compound ATP in MM patients compared to that seen in matched healthy individuals. Since ATP seems to work through purinergic 2 (P2) receptors, as a substance that evokes the exercise pressor reflex [51,58,83] by sensitizing group III mechanoreceptors and stimulating group IV metaboreceptors, the increased level of circulating ATP near contracting muscle could be a potential mediator of increased blood flow to working muscle in MM patients.

The test subject performs two exercise bouts with rhythmic submaximal handgrip contractions (40 pulls/min until exhaustion followed by a three-minute recovery phase. During the second bout, a forearm cuff is inflated to >30 mmHg above systolic arterial pressure inducing a circulatory clamp 10 s before end-exercise and during the recovery phase. The ergoreflex sensitivity (ES) is calculated as the percentage of the ventilation rate (VR) response to exercise maintained by circulatory clamp during the third minute of recovery (VR_2_) relative to the third minute of basal recovery (VR_1_). IV: Metaboreceptors (group IV afferents)

#### 4.1.5. Whole-Body Exercise: Lactate Turnover

Patients with MM are associated with lactic acidosis, sometimes even at rest, and resting lactate levels correlates closely with mtDNA mutation load in skeletal muscle of patients with MM [2]. Patients with MM have a low anaerobic threshold, which can be measured as a high respiratory exchange ratio (RER) during steady state exercise compared to healthy individuals. Therefore, many studies have tried to design the optimal exercise test to exploit the impaired oxidative capacity that translates into increased lactate levels in MM patients [19,20,21,24,26,76,81,84,85,86,87,88,89,90,91,92]. The most widely used test is the cycling exercise test with incremental workload to exhaustion (maximal cycle test) measuring peak-exercise lactate levels, but since many other non-neuromuscular (ischemic heart disease, lung disease, anemic patients) [93,94] and neuromuscular disorders [95] may also have exaggerated lactate during exercise, submaximal exercise test performed at a constant workload with measurement of plasma lactate has been introduced. During submaximal exercise, the relationship between lactate production and relative workload is well controlled and is, at least during a fixed exercise duration and at workload level well below the anaerobic threshold, less dependent on psychological (the will to continue) and physiological factors (fitness level) that determines the time that a person exercise above the anaerobic threshold and therefore to a large extend reflects the lactate level produced. Thus, in order to compare lactate levels, it might be more relevant to compare lactate level at the same workload after the same exercise duration when evaluating the metabolic state and when monitoring efficacy in treatment trials of patients with MM [12,25,91,95,96,97,98,99].

#### 4.1.6. Whole-Body Exercise: Diagnostic Yield

Since VO_2max_ is low and correlates directly with mtDNA mutation in skeletal muscle in patients with MM [2,3,7,10], it could be speculated that exercise testing with measurement of VO_2max_ could be a diagnostic tool for patients suspected of MM. However, patients with any kind of muscle weakness will present with low VO_2max_ and patients with other metabolic myopathies such as myophosphorylase and phosphofructokinase deficiencies can have a VO_2max_ comparable to that of patients with MM, which in these patients is the result of limited substrate delivery to the tricarboxylic acid (TCA) cycle [15,86,100,101,102,103,104]. Although VO_2max_ is an important indicator of oxidative capacity in patients with MM, VO_2max_ cannot be used as a single diagnostic measure for MM (Table 1). The exaggerated ventilator response to exercise that is a prominent symptom of MM could also potentially be used as a diagnostic marker for MM. However, just like VO_2max_ this symptom is found in conditions like heart and lung disease and even with age and in sedentary individuals [105] (Table 1). Along with the increased ventilation rate, the hyperkinetic circulatory response found in patients with MM has been found to be just as pronounced in patients with myophosphorylase and phosphofructokinase deficiencies [70,103,104,106] (Table 1). Thus, exaggerated ventilatory and circulatory responses to exercise may be characteristic, but not specific to MM (Table 1). Whether the potential underlying mechanism, the sensitized ergoreflex, could be a diagnostic screening target for MM is yet unknown. Though the test is described as simple and sensitive for MM [74], the method has not been studied in other neuromuscular conditions and therefore it remains unclear if ergoreflex hyperactivity is an exclusive feature of MM.

The increased resting lactate levels found in patients with MM, can be increased in other conditions, such as thiamin deficiency, which is a common condition (Prevalence 2:100,000 in western countries and up to 2000:100,000 in south-east Asian countries) [107]. Thus, high resting plasma lactate levels may be sensitive to, but not specific to MM. Studies have suggested that the low anaerobic threshold in MM patients could be exploited for diagnostic purpose during exercise testing and many studies with the purpose to design the optimal diagnostic test for MM with measurement of lactate have been conducted [12,19,20,21,24,25,26,76,81,84,85,86,87,88,89,90,91,98,99,106]. Studies have demonstrated that measurement of lactate levels during a submaximal exercise test versus a maximal exercise test is diagnostically superior for MM [20,91,95,96]. This may be true since lactate levels during a maximal exercise test is determined by how long a subject endures exercising above the anaerobic threshold, and since sedentary subjects, like patients with MM, who are unfamiliar with exertional activities stop exercising before reaching their true maximal exercise capacity. Moreover, dysfunctional ergoreflex activity driving autonomic dysfunction, including exaggerated ventilatory- and circulatory responses, may induce premature fatigue, further limiting the peak-exercise lactate levels. The notion that MM patients often do not reach their true maximal exercise capacity is derived from the consistent finding of lower maximal heart rate at peak exercise in patients with MM compared to age- and gender-matched sedentary subjects [108,109]. However, even though cycle testing at a constant workload with measurement of lactate levels may be diagnostically more sensitive for MM than a maximal exercise test, the diagnostic strength is not higher than a simple measurement of resting plasma lactate [95]. Thus, whole-body exercise with measurements of plasma lactate should not be routinely performed in the diagnostic workup of patients suspected of mitochondrial disease (Table 1).

#### 4.1.7. Whole-Body Exercise: GDF-15 as a Diagnostic Biomarker

During the last decade, there has been an increasing interest in Growth and Differentiation Factor 15 (GDF-15) as a possible biomarker of mitochondrial function [110,111,112,113,114,115,116,117,118]. GDF-15 belongs to the transforming growth factor beta super family of growth factors that regulate inflammation and apoptosis in injured tissue [118]. It has proven to be helpful in diagnosing mitochondrial disorders and can assist in the identification of MM from metabolic myopathies and muscular dystrophies [116]. However, serum GDF-15 is also increased in relation to stress responses, such as heart failure or lung disease. A one-hour cycling test increases GDF-15 in healthy individuals, suggesting GDF-15 increases with oxidative stress [118]. Interestingly, we have found that a maximal exercise test, which is of a significantly shorter duration than a one-hour exercise test and thus induces less oxidative stress in healthy controls, induces a five-fold increase in serum GDF-15-levels in patients with MM but induces no change in healthy individuals or in patients with metabolic myopathy [118]. Combining the biomarker with maximal exercise testing could therefore potentially enhance the diagnostic specificity, but further investigations are needed.

### 4.2. One-Extremity Exercise

#### 4.2.1. One-Extremity Exercise: Oxygen Delivery-Extraction and Oxidative Capacity

Measurement of oxygen saturation during a simple forearm, aerobic exercise test of 3 min at approximately 50% of maximal handgrip force is both sensitive and specific when screening for MM (Table 1). This is based on the finding that patients with MM have an impaired oxygen extraction from blood during muscle exercise. Oxygen extraction can be measured as either oxygen saturation in venous return from exercising muscle or indirectly by measuring the level of oxyhemoglobin with near infrared spectroscopy. Three studies demonstrated that measurement of oxygen saturation in blood from contracting muscle is both sensitive and specific [10,119,120], while near infrared spectroscopy seemed to be sensitive but not specific for mitochondrial myopathy [121,122,123,124]. These studies demonstrated that the high oxygen saturation was a result of impaired extraction alone and not increased oxygen delivery [10,11]. An important caveat to be aware of when conduction such tests, is that patients must have high venous baseline oxygen saturation levels. If the tested arm is cold, the veins are constricted, and the venous oxygen saturation will be very low which exerts a floor on the measurement of oxygen extraction. Another method that can be used as an indirect measurement of oxidative capacity is ^31^Phosphorous magnetic resonance spectroscopy (^31^P-MRS) [125,126,127]. This technique allows real-time, repeated and non-invasive assessment of muscle metabolism. The rate of ATP restoration and phosphocreatine (PCr) recovery after exercise almost exclusively rely on the oxidative capacity of the muscle. Thus, inorganic phosphorus (Pi), ADP and indirectly calculation of pH and lactate can be used as markers of anaerobic metabolism in skeletal muscle. Although these parameters can be used as indicators of mitochondrial function in skeletal muscle and ^31^P-MRS may serve as an interesting tool in follow-up in treatment trials, ^31^P-MRS cannot be used as a diagnostic tool for MM. This is true since the specificity for MM is low because many other neuromuscular disorders also demonstrate perturbed oxidative metabolism [104,105] (Table 1). We demonstrated that the diagnostic sensitivity at its best was 85% while the specificity was <65% [128]. Taking into consideration that ^31^P-MRS is time-consuming, not available in all neuromuscular centers and that both the test and the following data analysis requires specific training, ^31^P-MRS does not have a place in the diagnostic work-up of patients suspected of MM.

#### 4.2.2. One-Extremity Exercise: Lactate

Since 1951, a maximal forearm exercise test with measurements of plasma lactate has been used to identify inborn errors of muscle glycolysis and glycogenolysis as this test maximally stimulates glycolysis [129]. In line with this, maximal stimulation of oxidative phosphorylation during forearm exercise could reveal impaired oxidative capacity by increased levels of lactate. However, studies that have measured venous lactate directly [10,130], or indirectly using ^31^P-MRS [128], are not able to demonstrate higher levels of lactate during one-legged exercise in patients with MM. Although high lactate levels during exercise is indicative of impaired oxidative capacity, measurement of lactate levels during one-limb exercise cannot be used to diagnose patients with mitochondrial myopathy.

## 5. Outcome Measures

Though whole-body exercise testing cannot be used directly as a diagnostic tool, it has proven useful to track individual changes in response to interventions. With emerging pharmaceutical therapies under investigation for MM, the need for robust and clinically meaningful outcome measures is compelling. It is difficult to measure changes in mitochondrial dysfunction over time in liver and brain, but in skeletal muscle, changes in oxidative capacity are reflected by changes in exercise measures of VO_2_, ventilation rate, oxygen and metabolite turnover. These measures can therefore potentially serve as endpoints in clinical trials.

### 5.1. Maximal Exercise Testing

Whole-body exercise testing with the intent of reaching peak exercise has been used to identify exercise-induced improvement in oxidative capacity in MM patients (Table 1). These studies have used VO_2max_ in order to test changes in oxidative capacity with the intent of testing peak VO_2_ as an outcome measure. As already touched upon, VO_2max_ depends on both physiological and psychological factors, such as the will to continue and pain threshold. Even familiarization to the test has a great impact on if a subject reach peak VO_2_ and thus VO_2_ measure can be accounted for “true” VO_2max_. Thus, parameters like VO_2_ and plasma lactate levels cannot be used as endpoints unless the subject reaches maximal volition in both the pre- and post-intervention test for direct comparison. One of the simple parameters that can be used to ensure this is that the same heart rate level is reached, and that the slope of VO_2_ levels off just before the subject stops exercising.

### 5.2. Submaximal Exercise Testing

Carrying out exercise tests, investigating subjects at submaximal workload levels at a fixed exercise duration is less dependent on physiological parameters such as cardiac and pulmonary restrictions and moreover, depends less on physiological parameters such as the will to continue. If workload and pedaling cadence are kept the same between tests that are being compared pre- and post-intervention values can be directly compared. Changes in mitochondrial oxidative capacity can be reflected as changes in measures such as plasma lactate, heart rate and VO_2_ or energy expenditure measured with a metabolic cart or ^31^P-MRS in a one-legged model during constant workload exercise. These parameters have been used as measures of efficacy in studies on nutritional supplements (Q10, nitrate or creatine) targeted to increase the respiratory chain flux [131,132,133,134], or nutritional vasodilating components (L-arginine) [135] or anti-inflammatory agents (Omaveloxolone) [136] where whole-body exercise testing as measures of treatment responses were used. Ongoing clinical trials are using submaximal exercise parameters to assess the efficacy of the anti-oxidant resveratrol (NCT03728777) and L-arginine induced vasodilation (NCT01603446). In order to obtain useful measures of oxidative function from a submaximal exercise test, it is important to carefully consider the chosen workload with specific consideration of anaerobic threshold in relation to workload. A workload far below the anaerobic threshold will not stress the oxidative metabolism enough to reveal any intervention-related changes and a workload too high above the anaerobic threshold will potentially prematurely exhaust the patient, and thus potentially mask potential improvements. Most studies have addressed this issue by first measuring the patients VO_2max_, letting the patient exercise at 60 to 65% of VO_2max_ at either fixed duration or until exhaustion. From here, two different approaches can be made: either letting the subject exercise at the same pre-interventions workload or let the subject exercise at the new workload corresponding to the new 65% of VO_2max_. The duration until when the patient reaches anaerobic threshold, reaches exhaustion should be longer post intervention compared to pre-intervention, and heart- and ventilation rate and plasma lactate relative to workload should be lower if improvement in oxidative capacity.

## 6. Physical Fatigue

Physical fatigue can be defined as intolerance to sustain a muscle contraction or a level of workout during aerobic exercise and can be influenced by multiple factors including psychological issues (including the will to continue), CNS lesions, pain and fitness level. Which factors exactly induce fatigue in healthy individuals depend on the exercise modality. Thus, on a single-muscle level, fatigue is induced by physiological factors like restricted ATP production due to increased anaerobic metabolism, depletion of muscle glycogen and phosphocreatine stores along with increased lactate and free radical production [137]. On a whole-body level, fatigue may be induced by factors including impaired oxygen uptake combined with increasing carbon dioxide production and increasing lactate levels [137]. Fatigue is the most common patient-reported symptom in patients with MM [5,138]. In healthy individuals, a decrease in force production during maximal exercise can be regarded as a safety mechanism, since fatigue occurs before tissue damage, but in patients with mitochondrial disease, the sense of fatigue seems to occur before most patients reach maximal effort and does not seem to be related to a “safety-before-harm” mechanism. Lactate has been considered an end-product that potentially through hydrogen ion accumulation plays a role in the induction of fatigue [139]. It has been hypothesized that the high lactate levels relative to the low workload could induce the premature fatigue and exercise intolerance [12,85,88]. However, placebo-controlled studies lowering plasma lactate with dichloroacetate treatment were not able to find a positive effect on exercise capacity nor exercise tolerance in patients with mtDNA mutations [14]. This finding indicates that the premature fatigue seen in these patients is not related to lactate accumulation. In fact, we have shown, that lactate is an important fuel for exercising muscle in patients with MM to the same extent as in healthy individuals [80], and that the high lactate levels found in patients with MM are the result of a skewed lactate production/oxidation in the initial phase of exercise. This notion has been substantiated by findings of similar levels of lactate, pyruvate and other metabolites using microdialysis technique during exercise in patients with MM compared to matched healthy subjects [140], indicating that differences in reported fatigue during and after exercise is not related to lactate levels. We have previously suggested that premature fatigue could relate to the continuous recruitment of muscle fibers during sustained muscle work since some skeletal muscle fibers may harbor a high mtDNA mutation load while others have a low load [141]. Patients with MM must recruit more muscle fibers compared to healthy subjects exercising at the same workload since fibers with a high mtDNA mutation load rely on anaerobic glycolysis to generate ATP and therefore will quickly deplete stores of glycogen and creatine phosphate during exercise, and to sustain workload, more fibers has to be recruited. Findings of continuous lactate release along with substantial lactate oxidation during exercise in patients with mitochondrial myopathy support this notion [80].

Unlike heart rate, oxygen uptake and cardiac uptake, ventilation rate is strongly associated with perceived exertion during exercise [142,143]. During exercise, diaphragm and abdominal muscles are susceptible to fatigue since respiratory muscles compete with limb locomotor muscles for blood flow where blood flow seems to be redirected to limb muscles inducing fatigue of the respiratory muscles. Thus, studies have found evidence that respiratory muscle fatigue may be directly involved in inducing exercise intolerance [142,143]. The major consequence of respiratory muscle fatigue is an increased sympathetic vasoconstrictor outflow to working skeletal muscle through a respiratory muscle metaboreflex, thereby reducing limb blood flow and increasing the severity of exercise-induced locomotor muscle fatigue. Thus, although many forward and backward feedback mechanisms are yet to be investigated in patients with MM, the exaggerated ventilation rate in patients with MM may be an important factor in the perceived pre-mature fatigue and could be coupled to the exaggerated ergoreflex activity in patients with MM [74].

## 7. Exercise Training

With the current lack of effective treatment for MM, studies have focused on improving muscle function by exercise training [82,144,145,146,147,148,149,150,151,152,153,154,155,156]. In MM, this strategy has a specific effect on the disease as mitochondrial volume increases with exercise. For patients with mtDNA mutations, also the wild-type fraction of mitochondria will expand and thus help to widen the metabolic bottleneck. The many studies looking at effects of exercise training on oxidative capacity and mtDNA mutation load in muscle have been described extensively in previous reviews and in a meta-analysis on training effects in patients with neuromuscular disorders [157]. In short, the conclusion is that both aerobic and resistance training improve biochemical and physiological parameters in patients with MM [82,144,145,146,147,148,149,150,151,152,153,154,155,156,158]. Despite improving the maximal effort at peak exercise, studies have shown that improvement in maximal oxidative capacity aerobic exercise training improve maximal oxygen uptake and increased maximal oxidative capacity results in increased ability to extract oxygen from blood to contracting muscle, and improved maximum rate of ATP synthesis [148,149]. Additionally, Porcelli et al. demonstrated MM patients with impaired pulmonary VO_2_ pre-training, pulmonary VO_2_ kinetics was significantly increased with training resulting in reduced O_2_ deficit and higher exercise tolerance after 12 weeks of moderate intensity cycle training [150]. These improvements translate into increased tolerance during exercise at submaximal workload, with longer exercise duration along with lower lactate, lower heart rate and lower Borg scale level at the same workload pre- and post-training. Together with an overall finding of improved quality of life supported by patient self-assessed quality of life with respect to physical capacity and general well-being outcome measures from submaximal exercise tests demonstrate that physical activity is better tolerated after exercise training. Newman et al., is, to our knowledge, the first that has evaluated the sensitivity and specificity of clinician-rated outcome measures, i.e., 10 m walk test (10MWT), Timed up and Go (TUG) and the 5 times sit to stand (5XSTS) as primary outcome measures in evaluation of the effect of exercise training in patients with MM [159]. The authors demonstrated that the tests were able to demonstrate significant changes in physical capacity and interestingly, the 5XSTS test was able to discriminate between patients and healthy subjects with exercise intolerance due to sedentary lifestyle [159], indicating that test like TUG and 5XSTS may potentially be used as primary outcome measures instead of more advanced exercise tests, such as cycle tests.

Only one study has investigated long-term effect of aerobic training in four patients with different mtDNA mutations [146]. Whether exercise training is beneficial in the long term is yet to be investigated in a larger cohort. With aerobic training, patients with MM improve peak oxidative capacity measured with maximal exercise testing. It is yet unknown, whether this is a result of improved mitochondrial function or rather the circulatory and structural muscle adaptations that follow the change from a sedentary to an active lifestyle, but exercise training indisputably reduces functional limitations in patients with MM.

## 8. Perspectives

Exercise testing has been used to assess oxidative capacity in patients with MM even before genetic analyses were available. As described, differences between healthy subjects and patients with impaired oxidative capacity due to mtDNA mutation are found in many steps of oxygen transport and consumption. This includes abnormal ventilation rates, reduced maximal oxygen uptake, disturbed oxygen delivery, likely caused by dysregulated autonomic function and skewed oxygen extraction, and metabolite utilization and production. Despite a very limited role in the diagnostic workup, exercise testing can serve as an important tool to describe, quantify and follow up on the degree of oxidative impairment in patients with MM associated with mtDNA mutations, and thus serve as a surrogate marker of effect in interventional trials.

## Figures and Tables

**Table 1:** Summary of the exercise tests and their characteristics in patients with Mitochondrial Myopathy (MM) and their potential as diagnostic tools or as outcome measures in clinical trials.

Test	Measure	Method	Characteristics in MM	Diagnostic Potential	Strengths	Weaknesses
Maximal exercise	VO_2max_	CPET or Douglas bag	↓	**÷** ↑ Sensitive ↓ Specific	Equipment available in most hospitals. Directly reflects aerobic energy metabolism. Correlates to mtDNA mutation load.	Requires trained staff Sensitive to day-to-day variations in motivation to reach maximal exercise capacity
	VE/VO_2max_	CPET or Douglas bag	↑	**÷** ↑ Sensitive ↓ Specific		
	CO/VO_2max_	* Acethylene rebreathing	↑	**÷** ↑ Sensitive ↓ Specific	Corrects for circulatory adaptations affecting VO_2_-measurements during exercise	Requires specialized equipment
	Epinephrin/ Workload	Maximal exercise plasma value	↑	**÷** ↑ Sensitive ↓ Specific	Standard analysis in most hospitals.	Not specific for MM
	Plasma Lactate	Post- exercise sampling	↑	**÷** ↑ Sensitive ↓ Specific	Standard analysis	Sensitive to degree of volition to reach maximal effort. Equally specific to resting lactate
	Serum GDF-15	24h post-exercise sample	↑	**+** ↑ Sensitive ↓ Specific	Correlates with oxidative capacity	Not standard analysis. Further research in the use as outcome measure is required
Submaximal exercise	Plasma Lactate	Sampling during exercise	↑	**÷** ↑ Sensitive ↓ Specific	Easily standardized Can reflect changes in oxidative capacity	Requires prior maximal exercise testing. Workload must be selected carefully
	Heart rate	During exercise	↓	**÷** ↑ Sensitive ↓ Specific		
One-extremity exercise	ATP turnover	* ^31^P-MRS One-legged exercise	↓	**÷** ↑ Sensitive ↓ Specific	Indirect real-time measure of oxidative capacity in the tested extremity	Requires specialized equipment, and highly trained staff
	Oxygen saturation/ content	* Blood gas analyzer/ near infrared spectroscopy	↓	**+** ↑ Sensitive ↑ Specific	Correlates with mtDNA mutation load.	Test arm must be sufficiently warm to ensure venous blood flow
	Ergoreflex sensitivity	Handgrip +/−ischemia	↑	**÷** ↑ Sensitive ↓ Specific	Simple test setup. Correlates to degree of cardiac affection	Specificity vs. other myopathies needs to be investigated

↑: High, ↓: Low, +: Yes, **÷**: No, NA: Not applicable, CPET: Cardiopulmonary exercise testing, VO_2max_: Maximal oxygen uptake, VE: Ventilation rate, CO: Cardiac output, GDF-15: Growth and Differentiation Factor-15, AV: Arterio-venous, ^31^P-MRS: ^31^Phosphorous magnetic resonance spectroscopy, mtDNA: Mitochondrial DNA; * Requires specialized equipment and specialized/trained personal.
